# ^13^C magnetic resonance spectroscopy measurements with hyperpolarized [1‐^13^C] pyruvate can be used to detect the expression of transgenic pyruvate decarboxylase activity in vivo

**DOI:** 10.1002/mrm.25879

**Published:** 2015-09-21

**Authors:** Piotr Dzien, Sui‐Seng Tee, Mikko I. Kettunen, Scott K. Lyons, Timothy J. Larkin, Kerstin N. Timm, De‐En Hu, Alan Wright, Tiago B. Rodrigues, Eva M. Serrao, Irene Marco‐Rius, Elizabeth Mannion, Paula D'Santos, Brett W. C. Kennedy, Kevin M. Brindle

**Affiliations:** ^1^ Department of BiochemistryUniversity of CambridgeCambridgeUK; ^2^ Cancer Research UK, Cambridge Research Institute, Li Ka Shing CentreCambridgeUK.; ^3^ Present address: A. I. Virtanen Institute for Molecular Sciences, University of Eastern FinlandNeulaniementieKuopioFinland.

**Keywords:** reporter genes, *Zymomonas mobilis* pyruvate decarboxylase, hyperpolarized [1‐^13^C] pyruvate

## Abstract

**Purpose:**

Dissolution dynamic nuclear polarization can increase the sensitivity of the ^13^C magnetic resonance spectroscopy experiment by at least four orders of magnitude and offers a novel approach to the development of MRI gene reporters based on enzymes that metabolize ^13^C‐labeled tracers. We describe here a gene reporter based on the enzyme pyruvate decarboxylase (EC 4.1.1.1), which catalyzes the decarboxylation of pyruvate to produce acetaldehyde and carbon dioxide.

**Methods:**

Pyruvate decarboxylase from *Zymomonas mobilis* (*zm*PDC) and a mutant that lacked enzyme activity were expressed using an inducible promoter in human embryonic kidney (HEK293T) cells. Enzyme activity was measured in the cells and in xenografts derived from the cells using ^13^C MRS measurements of the conversion of hyperpolarized [1‐^13^C] pyruvate to H^13^ ${\text{CO}}_{3}^{–}$.

**Results:**

Induction of *zm*PDC expression in the cells and in the xenografts derived from them resulted in an approximately two‐fold increase in the H^13^ ${\text{CO}}_{3}^{–}$/[1‐^13^C] pyruvate signal ratio following intravenous injection of hyperpolarized [1‐^13^C] pyruvate.

**Conclusion:**

We have demonstrated the feasibility of using *zm*PDC as an in vivo reporter gene for use with hyperpolarized ^13^C MRS. Magn Reson Med 76:391–401, 2016. © 2015 The Authors. Magnetic Resonance in Medicine published by Wiley Periodicals, Inc. on behalf of International Society for Magnetic Resonance in Medicine. This is an open access article under the terms of the Creative Commons Attribution License, which permits use, distribution and reproduction in any medium, provided the original work is properly cited.

## INTRODUCTION

Reporter genes provide a means of visualizing gene expression in living organisms. The introduction of fluorescence and bioluminescence reporter genes has revolutionized molecular cell biology, to a large extent due to the relative simplicity and speed of these imaging modalities. However, despite good sensitivity, the use of these techniques is restricted in vivo due to poor depth penetration, which limits their use in superficial tissues. Reporter genes developed to work with positron emission tomography have very good depth penetration and sensitivity but have limited spatial resolution and may require an on‐site cyclotron for probe synthesis 1, whereas MR reporters can have good spatiotemporal resolution and depth penetration but may be limited by a lack of sensitivity. Several systems have been developed that use T_1_ and T_2_ (*) MRI contrast agents [reviewed in Lyons et al. 1 and Patrick et al. 2]. However, these can suffer from background signal, as water proton relaxation rates may change independently of reporter gene expression, for example due to hemorrhage, changes in vascularity, or the development of air cavities and calcifications 3. Furthermore, phagocytosis by infiltrating macrophages of dead cells that contain contrast agents can give rise to persistent nonspecific contrast 3. In addition, high magnetic fields may be required to achieve sufficient contrast with some agents that modulate T_2_/T_2_* relaxation rates 4.

The first enzyme‐based magnetic resonance spectroscopy (MRS) gene reporter was creatine kinase, which was expressed in *Escherichia coli* 5, yeast 6, and murine liver 7, where the activity of the enzyme was detected by ^31^P MRS. This approach exploited the fact that creatine kinase is not expressed endogenously in these systems; however, the low sensitivity of ^31^P MRS limited the temporal, and in the liver, spatial resolution.

There are several examples of ^19^F‐based gene reporters, where the reporter is an enzyme that catalyzes transformation of a fluorinated substrate. ^19^F MR‐based reporters have the advantage that there is effectively no background signal in biological systems, the nucleus has a broad chemical shift range and it is relatively sensitive to MRS detection. The possibility of noninvasive monitoring of therapeutic gene expression in vivo using ^19^F MRS was demonstrated in xenografts stably expressing carboxypeptidase G2, a bacterial enzyme that mediates the release of DNA alkylating agents by enzymatic cleavage of labile pro‐drugs 8. The conversion of 3,5‐difluorobenzoyl‐l‐glutamic acid to 3,5‐difluorobenzoic acid was measured using ^19^F MRS and thus provided a readout of the activity of this therapeutic transgene within the target tissue 8. Another ^19^F MRS‐based reporter exploited the enzyme β‐galactosidase, encoded by the lacZ gene, which has been widely used as an optical reporter. β‐Galactosidase catalyzes the cleavage of 2‐fluoro‐4‐nitrophenyl β‐d‐galactopyranoside (OFPNPG) to aglycone 2‐fluoro‐4‐nitrophenol, which results in the appearance of a new resonance in the ^19^F spectrum 9. The expression of lacZ in human PC‐3 prostate carcinoma cells grown as xenografts in mice was detected by ^19^F MRS after intratumoral injection of OFPNPG 9.

The introduction of dissolution dynamic nuclear polarization, a method that can increase the sensitivity of ^13^C MRS by at least four orders of magnitude, has allowed imaging of some ^13^C labeled substrates and the products of their metabolism in real time in vivo 10. Reporter genes encoding enzymes that process hyperpolarized ^13^C‐labeled tracers would give rise to signal, which like ^19^F has no background and could be imaged within a few minutes. The short half‐life of the hyperpolarization and rapid clearance of small molecule metabolites means that reporter gene expression could be reinterrogated very rapidly. This is in contrast to reporters that use T_1_ or T_2_/T_2_* contrast agents, which may require several hours or days to clear 2, 11, which limits the temporal resolution of longitudinal assessment of gene expression using T_1_ or T_2_/T_2_*‐weighted imaging.

The enzyme‐based reporter genes that have been developed for use with hyperpolarized ^13^C‐labeled cell substrates are summarized in Table 1. However, a number of factors have prevented their successful implementation in vivo, including insufficient levels of polarization, a short T_1_ relaxation time of the hyperpolarized ^13^C label 12, low specific activity of the enzyme with the labeled substrate 13, and insufficient levels of enzyme activity in the expressing cells 12, 14.

**Table 1:** Enzyme‐based reporter genes developed for use with hyperpolarized ^13^C‐labeled cell substrates.

Reporter Gene Protein Product	Reporter Enzyme Hyperpolarized Substrate	Reporter Enzyme Hyperpolarized Product	Suggested Application	Reference
Carboxypeptidase G2	3,5‐difluorobenzoyl‐l‐glutamic acid (3,5‐DFBGlu)	3,5‐difluorobenzoic acid (3,5‐DFBA)	Gene‐directed enzyme prodrug therapies	12
Aminoacylase‐1	[1‐^13^C] *N*‐acetyl‐l‐methionine	[1‐^13^C] methionine	Monitoring longitudinal interaction between cell and host in cell‐based therapies	14
Maltose‐binding protein‐mouse lactate dehydrogenase fusion	[1‐^13^C] 4‐methyl‐2‐oxopentanoic acid	[1‐^13^C] 4‐methyl‐2‐ hydroxypentanoic acid	Protein tagging	13

**Figure 1:**
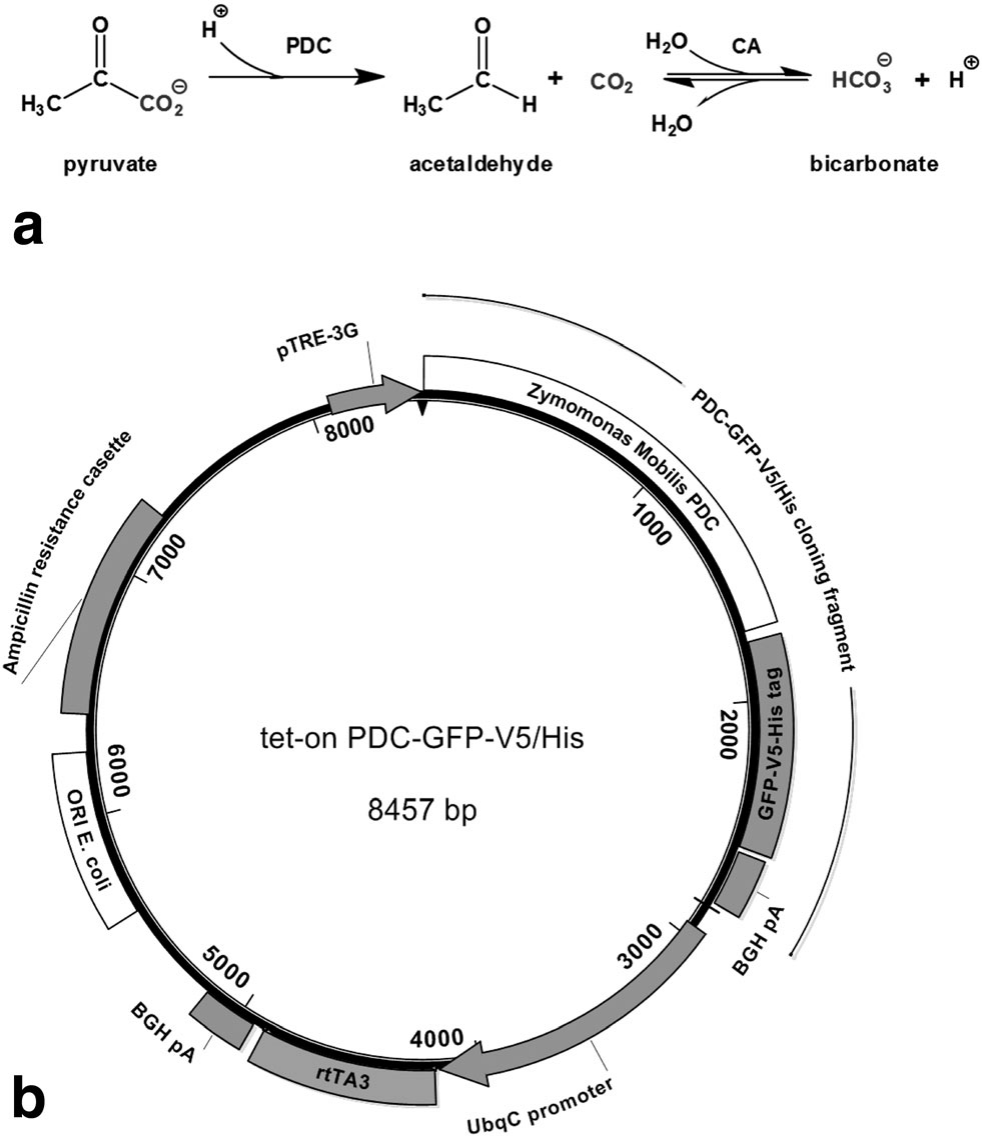
(a) Reaction catalyzed by *zm*PDC and subsequent fate of the carbon dioxide produced. CA, carbonic anhydrase. (**b**) Tet‐on PDC‐GFP‐V5/His vector map. BGH pA, bovine growth hormone poly A sequence (ampicillin resistance cassette contained β‐lactamase coding sequence); ORI E. coli, bacterial origin of replication; pTRE3G, third generation trans‐activator response element containing incomplete herpes virus promoter; rtTA3, trans‐activator protein; UbqC, constitutive mammalian ubiquitin C promoter.

We demonstrate here the use of PDC, from the bacterium *Zymomonas mobilis*, as an MR gene reporter in HEK293T human embryonic kidney cells 15, and in implanted xenografts that were derived from these cells. The PDC‐catalyzed decarboxylation of pyruvate to give acetaldehyde and carbon dioxide is a reaction that is not present in mammalian tissues and can be monitored using ^13^C MRS measurements of ^13^C bicarbonate production following injection of hyperpolarized [1‐^13^C] pyruvate (Fig. 1a). Labeled bicarbonate is produced by spontaneous and carbonic anhydrase–catalyzed exchange of the hyperpolarized ^13^C label between ^13^CO_2_ and the intracellular bicarbonate pool 16.

## METHODS

All chemicals were purchased from Sigma Aldrich (Gillingham, Dorset, UK), unless stated otherwise.

### Generation of Clonal HEK293T Cells Stably Transfected with Doxycycline‐Inducible Vectors Expressing Wild‐Type and Mutant PDC

A DNA sequence encoding a monomer of pyruvate decarboxylase from *Zymomonas mobilis* (*zm*PDC) fused to a green fluorescent protein (GFP) with V5 and histidine epitope tags in a pEF6‐V5/His vector (Life Technologies Ltd, Paisley, UK) was cloned by PCR as an *AgeI/FseI* fragment into a tet‐on doxycycline‐inducible vector (pBS‐TRE3G‐YFP‐ZnF‐rtTA3pA) to give tet‐on *zm*PDC‐GFP‐V5/His (Fig. 1). A variant of this plasmid, encoding an inactive H113Q point mutant of *zm*PDC 17, was made by cloning a synthetic gene fragment into the tet‐on *zm*PDC‐GFP‐V5/His plasmid (see Supporting Information Section S1) to give tet‐on *zm*PDC/H113Q‐GFP‐V5/His. HEK293T cells were grown at 37°C in Dulbecco's Modified Eagle's Medium (DMEM) (GIBCO Invitrogen, Carlsbad California, USA) with 10% fetal bovine serum (PAA Laboratories Ltd, Yeovil Somerset, UK), 4 mM glutamine (GIBCO Invitrogen) and 5% CO_2_. Plasmid DNA transfections were performed with Lipofectamine (Life Technologies Ltd) with a 10:1 molar ratio mixture of the tet‐on PDC plasmid and pPUR vector (Clontech Laboratories Inc., Mountain View, California, USA). Stably transfected clones were selected in media containing 2 μg/mL puromycin, picked, and expanded (see Supporting Information Section S2).

### Analysis of Cell Growth

Twenty‐five thousand cells were seeded per well in 48‐well plates (NUNC; Thermo Fisher Scientific, Waltham, Massachusetts, USA), with six replicates per experimental condition. Eight hours after seeding, an appropriate volume of a 1 mg/mL solution of doxycycline in sterile water, or water alone, was added to the media. Confluence of the monolayer was measured using an IncuCyte instrument (Essen Instruments, Essen, Michigan, USA), which was then used to calculate the cell doubling time using Prism 6.0 (GraphPad Software, Inc., La Jolla, California, USA) .

### Time Course of Doxycycline‐Induced PDC Expression

The growth medium was refreshed 24 h after cell seeding in T25 tissue culture flasks. For dose–response experiments, doxycycline was added to the media at the indicated concentrations, and the cells were harvested 48 h later. For time course experiments, the medium was supplemented with 2 µg/mL of doxycycline at 24, 48, or 96 h before cell harvesting.

### Cell Extraction and Western Blotting

Cells were lysed in passive lysis 5× buffer (Promega, Madison, Wisconsin, USA), diluted in water, and contained complete ethylenediaminetetraacetic acid–free protease inhibitor cocktail (Roche Applied Science, Basel, Switzerland) as recommended by the manufacturer. Protein concentration was measured using a Bradford protein assay and a Sunrise 96‐well plate reader (Tecan Group Ltd, Männedorf, Switzerland). The protein was separated by sodium dodecyl sulfate–polyacrylamide gel electrophoresis, blotted onto a polyvinylidene difluoride membrane, and probed for expression of the V5 epitope and α‐actin (see Supporting Information Section S3).

### ^13^C T_1_ Measurements In Vitro

Measurements were made using a 11.7 T Bruker Avance II^+^ 500 MHz spectrometer and an inversion recovery pulse sequence in samples containing the molecule of interest, 10% D_2_O and 4% bovine serum albumin with a pH between 7.4 and 7.6. Table 2 shows the sample composition and the delays in the inversion recovery sequence.

**Table 2:** Sample composition and the delays in the inversion recovery sequence used for T_1_ measurements in vitro.

Sample	^13^C Concentration (mM)	TR (s)	Delays	
			Number	Duration (s)
[1‐^13^C] pyruvate	10	220	16	0.5–200
[2‐^13^C] pyruvate	10	220	16	0.5–200
^13^C bicarbonate	20	240	16	1.0–240
[1‐^13^C] acetaldehyde	5	55	16	0.1–40

### Dynamic Nuclear Polarization

[1‐^13^C] or [2‐^13^C] pyruvic acid were polarized and dissolved as described previously 18. The dissolved samples contained 75 mM [1‐^13^C] or [2‐^13^C] pyruvate (see Supporting Information Section S4).

### ^13^C MRS Measurements with Hyperpolarized ^13^C‐Labeled Pyruvate In Vitro

Cells (5 × 10^7^) in 2 mL of pyruvate‐, and FBS‐free DMEM in a 10 mm NMR tube were placed in a vertical bore 9.4T NMR spectrometer with a broadband probe tuned to ^13^C (Varian NMR Instruments, Palo Alto, California, USA). Typically 9‐12 s after dissolution, 2 mL of the dissolved polarized sample containing 75 mM ^13^C‐labeled pyruvate were injected into the tube, and 180 spectra with a flip angle of 12° were acquired (data points = 5440, acquisition time = 0.170 s, repetition time [TR] = 0.25 s, number of transients = 4, sweep width = 16 kHz) at 37°C.

### ^1^H MRS Measurements with [U‐^2^H_3_] Pyruvate In Vitro

Sodium pyruvate was deuterated as described previously 19. Approximately 5.0 × 10^7^ cells were suspended in pyruvate‐free DMEM. Each cell suspension was split into two equal volumes, one of which was pelleted and frozen in liquid nitrogen. The remaining sample was resuspended in 0.54 mL of pyruvate‐free DMEM (containing 5 mM 2,2,3,3‐D4 sodium‐3‐trimethylsilylpropionate and 12.5% D_2_O) in a 5‐mm‐diameter NMR tube and placed in a vertical bore 11.7 T Bruker Avance II^+^ 500 MHz spectrometer. Two or three control spectra were acquired before 60 µL of a 0.25 M solution of sodium [U‐^2^H_3_] pyruvate in H_2_O was added to the cell suspension and mixed. After the addition of 100 μL of mineral oil, the sample was returned to the spectrometer and, after thermal equilibration, a series of 16 ^1^H spectra were collected using a pulse and acquire sequence with 90° flip angle pulses (data points = 16384, acquisition time = 1.0 s, sweep width = 8 kHz, number of transients = 3, TR = 39 s). The cell pellets were lysed in 0.45 mL of ice‐cold lysis buffer, essentially as described above, except that the lysis buffer was diluted in 200 mM citrate buffer (pH 6.0). A 480‐μL sample of the lysate was then mixed with 60‐μL of a solution of 10 mM thiamine pyrophosphate and 10 mM MgCl_2_ in ^2^H_2_0 and used for ^1^H MRS measurements.

### Animal Experiments

All animal experiments were performed in compliance with a project license issued under the Animals (Scientific Procedures) Act of 1986 and were designed with reference to the UK Coordinating Committee on Cancer Research guidelines for the welfare of animals in experimental neoplasia 20. Protocols were approved by the Cancer Research UK, Cambridge Institute Animal Welfare and Ethical Review Body. Six to eight week‐old female severe combined immune‐deficient (SCID) mice (Charles River Ltd, Margate, UK) were used in each experimental group.

### Xenograft Implantation and Induction of PDC Expression In Vivo

Each animal was injected subcutaneously in the left flank with 0.1 mL of a cell suspension containing 2.5 × 10^7^ cells stably transfected with the tet‐on PDC‐GFP‐V5/His plasmid expressing the wild‐type or mutant enzymes. Xenograft diameters were measured using calipers, and the length (L) and width (W) were used to calculate their volumes (V) according to the formula V = ½ (LW^2^) 21.

The xenografts typically reached a volume of approximately 0.5–0.7 cm^3^ within 21–25 days from implantation. To induce PDC expression, a solution of 10 mg mL^−1^ doxycycline with 5% w/v sucrose was given to the mice in their drinking water ad libitum for 96 h prior to imaging experiments.

### ^13^C MRS Measurements In Vivo

Animals were anesthetized and monitored as described previously 22. A 20‐mm‐diameter surface coil (Rapid Biomedical GmbH, Rimpar, Germany) was placed over the xenograft, and the entire assembly was placed in a ^13^C/^1^ H volume coil (Rapid Biomedical) in a 7T horizontal bore magnet (Varian). Xenografts were localized in transverse ^1^H images acquired using a spin‐echo pulse sequence (TR = 1.5 s; echo time = 10 ms; field of view = 40 × 40 mm; data matrix = 128 × 128; slice thickness = 2 mm; number of slices = 15). Immediately after dissolution, 10 µL/g body weight of the hyperpolarized pyruvate solution (typically 240 µL in total) was injected via the tail vein over a period of 2 s. Spectroscopic acquisitions were started 30 s from the injection. Single transient spectra (128 spectra with TR = 500 ms, nominal flip angle = 20°, sweep width = 6 kHz; the delay from the middle of the excitation pulse to the beginning of data acquisition was 900 µs) from an 8‐mm slice through the tumor were acquired. The center frequencies for the spectra were selected to be 175 ppm and 200 ppm for experiments with [1‐^13^C] pyruvate and [2‐^13^C] pyruvate, respectively. For experiments with [2‐^13^C] pyruvate, the protons were decoupled using a WALTZ decoupling sequence during acquisition (B1 ∼1.65 kHz), centered at 9.7 ppm.

### Tissue Extraction

Freeze‐clamped xenograft tissues were homogenized using a manual Potter S homogenizer (Sartorius, Epsom, UK) in 4 volumes (milliliters per gram wet weight of tissue) of ice‐cold lysis buffer. Homogenates were centrifuged at 16,200 *g* and 4°C in an Eppendorf 5415D bench top centrifuge (Eppendorf, Hamburg, Germany) to remove debris.

### Immunohistochemistry

Immunohistochemical protocols and image analysis were performed using instruments, reagents, and software from Leica (Leica Microsystems, Milton Keynes, UK) unless stated otherwise. Xenografts were excised and fixed in 4% neutral buffered formalin solution for 24 h and then transferred to 70% ethanol. After paraffin embedding, tissue sections were taken from the center of the xenograft and stained for cleaved caspase 3 (CC3), V5 epitope, and GFP expression. Hematoxylin and eosin (H&E) staining was performed to assess tissue viability (see Supporting Information Section S5).

### Image Analysis

Slides were digitized using a Leica Aperio XT120 and analyzed using Aperio ImageScope software. For analysis of H&E staining, the area covered by nonviable xenograft tissue (as judged by the loss of structural coherence and visible chromatin condensation) was identified manually and compared with the total xenograft area, and the result was converted to percentage cell death. The Aperio ImageScope Positive PixelCount v9 macro, tuned to identify negatively and positively 3,3’‐diaminobenzidine–stained tissue, was used for quantification of CC3 staining, which is reported as the number of positive pixels as a percentage of the total number of pixels.

### Statistical Analysis

The results are presented as the mean ± standard deviation unless stated otherwise. Statistical significance was assessed using Microsoft Excel with a two‐tailed *t* test at the 95% confidence level.

## RESULTS

^13^C T_1_s measured in vitro were 42.2 s for [1‐^13^C] pyruvate (n = 2; 42.4 s and 42.0 s), 29.5 s for [2‐^13^C] pyruvate (n = 2; 29.4 s and 29.5 s), 11.5 s for [1‐^13^C] acetaldehyde (n = 2; 11.2 and 11.7 s), and 40.7 s for bicarbonate (n = 2; 40.7 s and 40.6 s) at 11.7T (Table 2). Although these T_1_ values were measured at 11.7T, we expect them to be shorter (because of the effect of chemical shift anisotropy) than those measured at 9.4 T and 7 T, which were the magnetic field strengths used for the cell and xenograft experiments, respectively.

**Figure 2:**
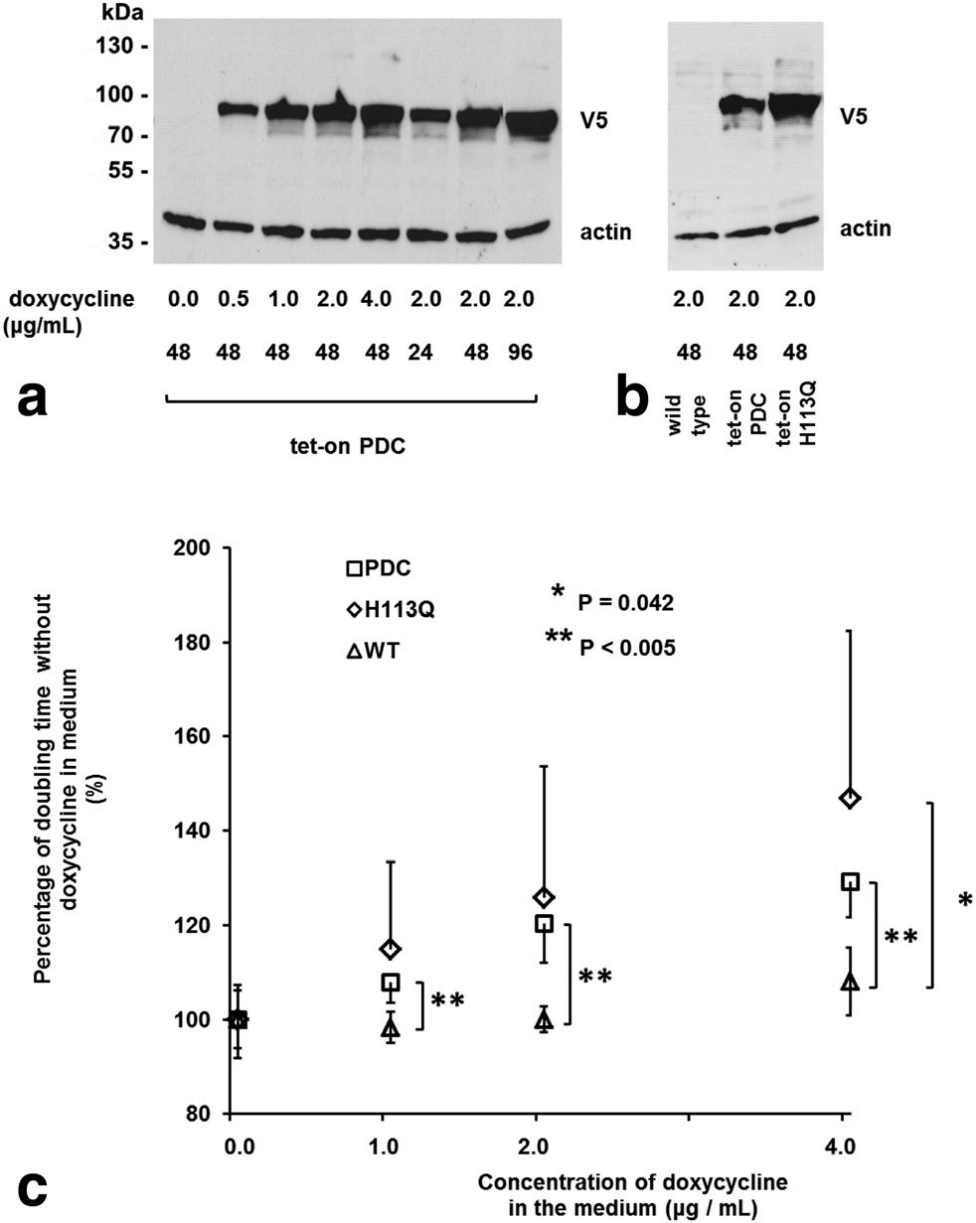
(a) Induction of PDC‐GFP‐V5/His protein expression in vitro. Western blot of a protein extract prepared from cells transfected with the tet‐on PDC‐GFP‐V5/His construct, grown with the indicated concentrations of doxycycline for the indicated times (h) and harvested when ∼80% confluent. A total of 20 µg of protein was loaded per well. Membranes were probed with antibodies against the V5 tag and actin (loading control). The typical exposure time of the x‐ray film was 10–15 s. (**b**) Western blot of a protein extract prepared from cells transfected with the tet‐on PDC‐GFP‐V5/His and tet‐on PDC/H113Q‐GFP‐V5/His constructs and harvested 48 h after the addition of 2 µg/mL doxycycline to the growth medium. (**c**) Effect of doxycycline‐induced PDC‐GFP‐V5/His and PDC/H113Q ‐GFP‐V5/His expression on cell doubling times. Four different clones stably transfected with the tet‐on PDC‐GFP‐V5/His construct and four clones transfected with the tet‐on PDC/H113Q ‐GFP‐V5/His construct were used. Untransfected HEK93T cells were used as controls. The data for each clone are an average of at least two independent experiments, each of which was seeded in six replicates. Error bars represent one standard deviation of the mean. Only positive or negative error bars are shown for clarity.

Expression of PDC in HEK293T cells transfected with the *zm*PDC‐GFP‐V5/His transgene was detectable on western blots by 24 h after addition of 2 μg/mL of doxycycline to the growth medium and showed further increases at 48 and 96 h. Expression was dose dependent, showing an increase between 0.5 and 2.0 μg/mL doxycycline and was undetectable in the absence of the drug (Fig. 2a). The H113Q mutant, which had no detectable PDC activity [data not shown and Huang et al. 17], showed greater doxycycline‐induced expression than the wild‐type enzyme (Fig. 2b).

The cell doubling times of wild‐type HEK293T cells, cells transfected with *zm*PDC‐GFP‐V5/His, and cells transfected with *zm*PDC/H113Q‐GFP‐V5/His increased after the addition of doxycycline to the growth medium in a dose‐dependent manner. At 4 µg/mL doxycycline, the doubling times of cells expressing the wild‐type and mutant enzymes were 129.4 ± 7.6% and 146.8 ± 35.6%, respectively, of their doubling times in the absence of doxycycline (Fig. 2c).

**Figure 3:**
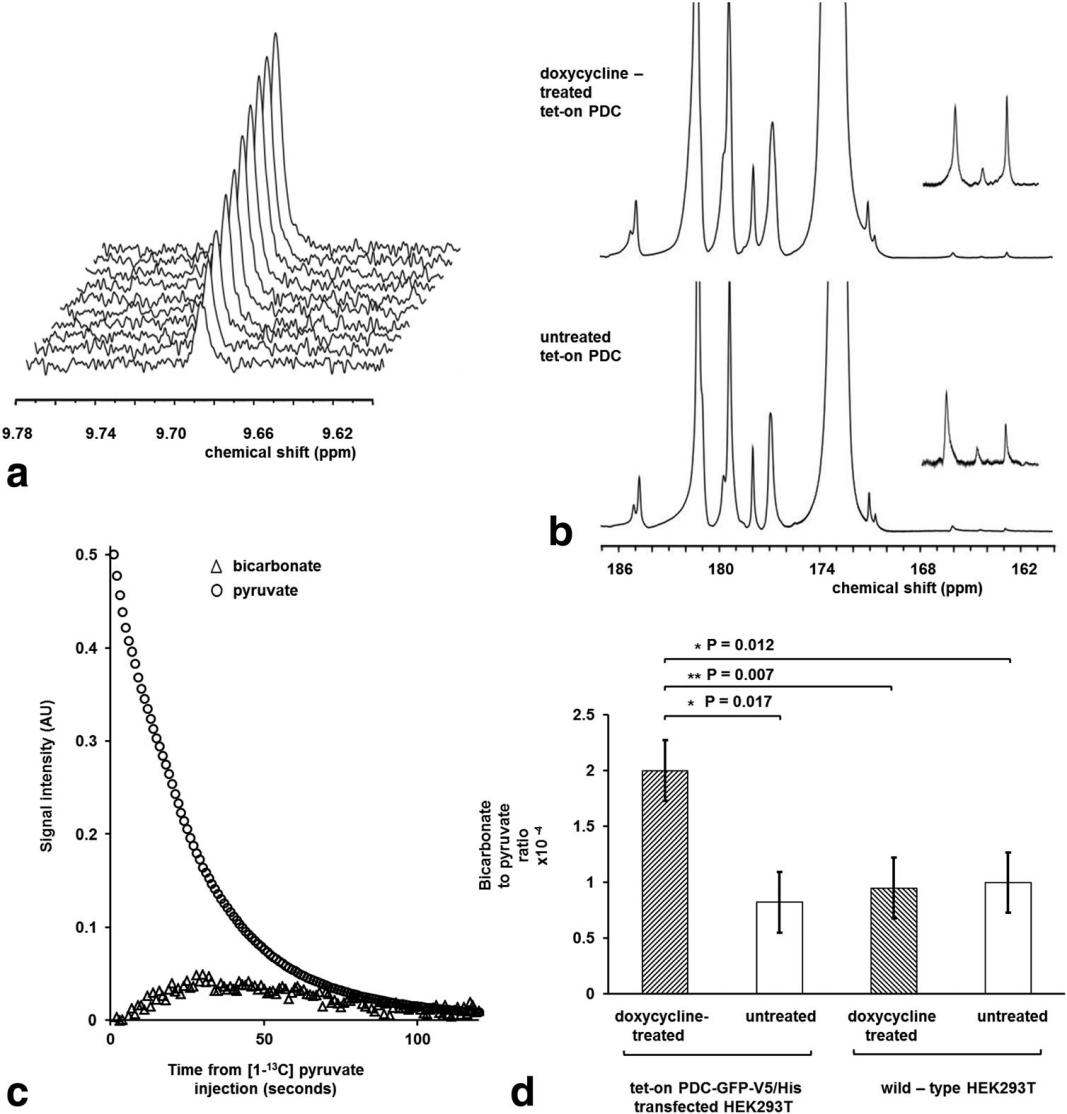
Detection of zmPDC activity in vitro following administration of [U‐^2^H_3_] pyruvate and hyperpolarized [1‐^13^C] pyruvate. (**a**) ^1^H spectra were acquired every 2 min, starting from ∼5 min after the addition of 25 mM [U‐^2^H_3_] pyruvate, to a lysate prepared from 2.5 × 10^7^ cells that had been grown with 2 µg/mL doxycycline in the medium for 48 h prior to harvest. (**b**) Spectra acquired during the first 80 s after addition of 75 mM hyperpolarized [1‐^13^C] pyruvate to suspensions of 5 × 10^7^ cells that had been transfected with the tet‐on PDC‐GFP‐V5/His construct. The cells had been grown with 2 μg/mL doxycycline in the medium for 48 h prior to experiments (doxycycline‐treated tet‐on PDC) or without doxycycline (untreated tet‐on PDC). Peak assignments: lactate (185 ppm), pyruvate hydrate (181 ppm), alanine (177 ppm), pyruvate (172.9 ppm), bicarbonate (162.8 ppm). The insets show a 15× vertical expansion of the 161–168 ppm region of the spectra. The ^13^C bicarbonate peak at 162.8 ppm was accompanied by unidentified resonances at 166 ppm and 164.3 ppm. (**c**) Pyruvate and bicarbonate signal intensities following the addition of 75 mM hyperpolarized [1‐^13^C] pyruvate to a suspension of cells expressing *zm*PDC. Measurements were made at 9.4T at 37° C (pH ∼7.0). The bicarbonate signal was multiplied 2000 times. (**d**) Average ratio of the bicarbonate to pyruvate signal intensities in panel b (n = 3 for all experimental conditions).

PDC activity in cells that had been transfected with *zm*PDC‐GFP‐V5/His and grown in the presence of 2 μg/mL doxycycline for 48 h was first assessed using ^1^H MRS measurements of [2,2,2‐^2^H_3_] acetaldehyde production following the addition of [U‐^2^H_3_] pyruvate (Fig. 3a). Lysed cells showed a 5.4‐fold higher acetaldehyde production rate compared with intact cells (5.6 × 10^−4^ ± 0.8 × 10^−4^ U/10^7^ cells and 1.1 × 10^−4^ ± 0.2 × 10^−4^ U/10^7^ cells [SD, n = 3], respectively), demonstrating that the activity of the enzyme in the cells was limited by the rate of pyruvate transport. PDC activity was not detectable in cells that did not express *zm*PDC‐GFP‐V5/His protein or that expressed *zm*PDC H113Q‐GFP‐V5/His protein at 48 h after addition of 2 μg/mL doxycycline to the growth medium [data not shown and Huang et al. 17]. We were unable to detect PDC activity in vivo in xenografts derived from cells expressing *zm*PDC‐GFP‐V5/His, using similar ^1^H MRS measurements, following intraperitoneal injection of [U‐^2^H_3_] pyruvate (see Supporting Fig. S1). Addition of hyperpolarized [1‐^13^C] pyruvate to cells resulted in a time‐dependent increase in the bicarbonate signal at 162.8 ppm, which could be followed using dynamic ^13^C MRS measurements (Fig. 3b). Doxycycline‐induced expression of the wild‐type enzyme resulted in an approximately 2.5‐fold higher ^13^C bicarbonate/pyruvate signal ratio calculated from the sum of first 80 spectra acquired after the addition of 75 mM hyperpolarized [1‐^13^C] pyruvate (Fig. 3c, 3d), when compared with uninduced cells or wild‐type cells incubated in the presence or absence of doxycycline.

**Figure 4:**
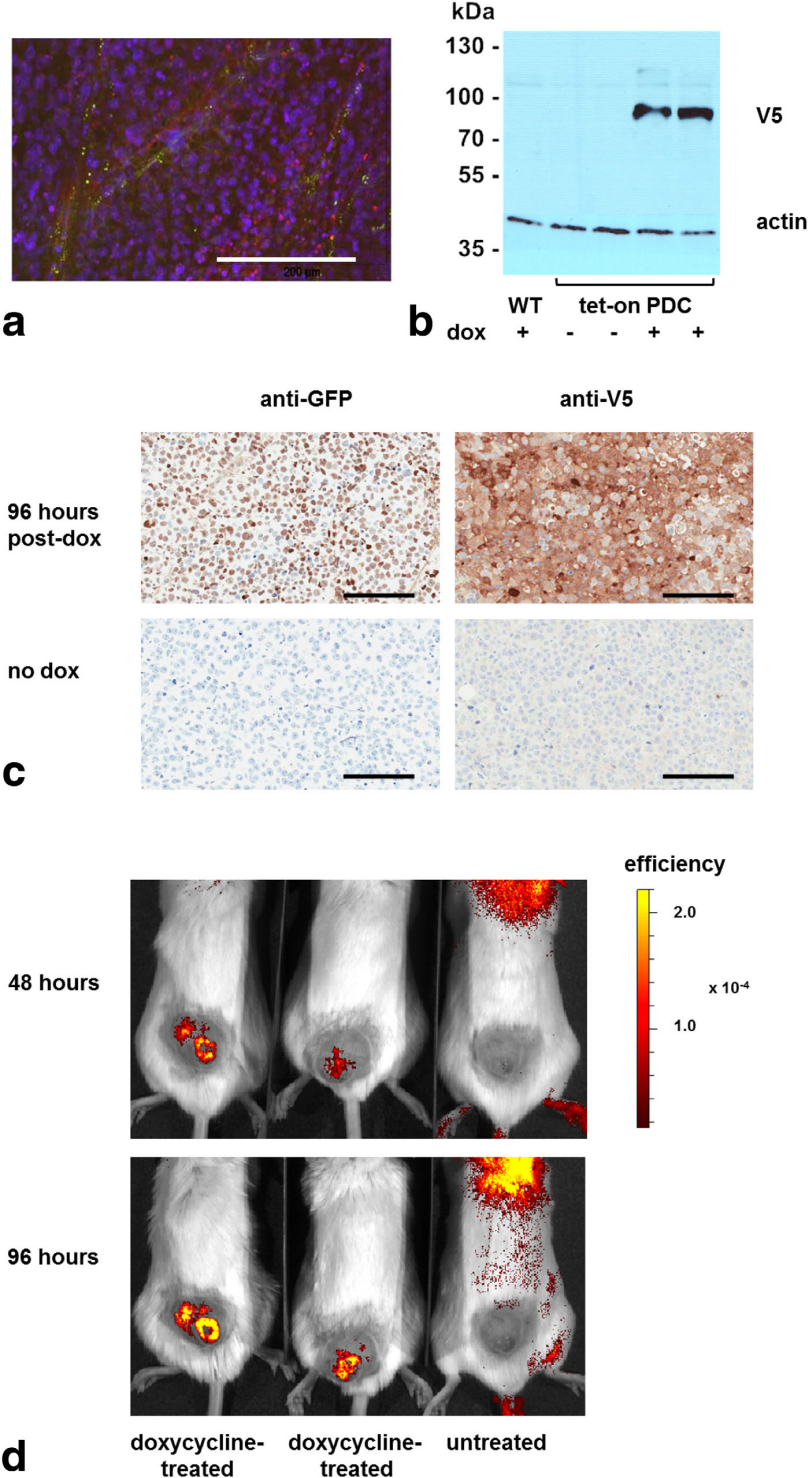
Demonstration of *zm*PDC protein expression in vivo in xenografts derived from HEK293T cells transfected with the tet‐on PDC‐GFP‐V5/His construct. (**a**) Fluorescence in situ hybridization of paraffin‐embedded sections of HEK‐293T–derived xenografts. Representative images of sections, stained simultaneously with mouse‐specific centromeric probes (green) and human‐specific centromeric probes (red), were acquired at 40× magnification. Nuclei were stained with DAPI (blue). Scale bar = 150 μm. (**b**) Western blot of protein extracts prepared from xenografts derived from HEK293T cells transfected with the tet‐on PDC‐GFP‐V5/His construct. Animals were given either no doxycycline (–) or 10 mg/mL doxycycline in their drinking water for 96 h prior to preparation of the protein extract (+). (**c**) Representative formalin‐fixed stained sections taken from xenografts grown for ∼ 21–24 d following subcutaneous implantation of cells transfected with the tet‐on PDC‐GFP‐V5/His construct. Where indicated, the mice were given 10 mg/mL doxycycline solution in their drinking water for 96 h prior to xenograft excision. PDC expression was investigated by probing the sections with anti‐GFP and anti‐V5 antibodies. Scale bars = 150 µm. (**d**) GFP fluorescence overlaid on bright field images acquired from a single cohort of mice at 48 and 96 h after the beginning of doxycycline administration. Ten mg/mL of doxycycline was given in the drinking water where indicated. Images were acquired with an IVIS 200 camera and were analyzed with Living Image software (both from Perkin‐Elmer). The post‐doxycycline GFP fluorescence images are representative of experiments on five animals. The tumors were located on the backs of the animals immediately above the tail (see Supporting Information Section S6).

Subcutaneous implantation of cells in SCID mice resulted in xenografts that consisted predominantly of HEK‐293T cells (Fig. 4a). Xenografts derived from cells transfected with the *zm*PDC‐GFP‐V5/His vector expressed *zm*PDC‐GFP‐V5/His protein at 96 h after doxycycline administration, as demonstrated by western blots of tissue extracts. The protein was not detectable in the absence of doxycycline administration or in xenografts derived from wild‐type HEK293T cells (Fig. 4b). *zm*PDC‐GFP‐V5/His protein expression was confirmed by staining of tissue sections with antibodies against GFP and the V5 epitope (Fig. 4c). Expression was also confirmed by imaging GFP fluorescence at 48 and 96 h after doxycycline administration (Fig. 4d).

**Figure 5:**
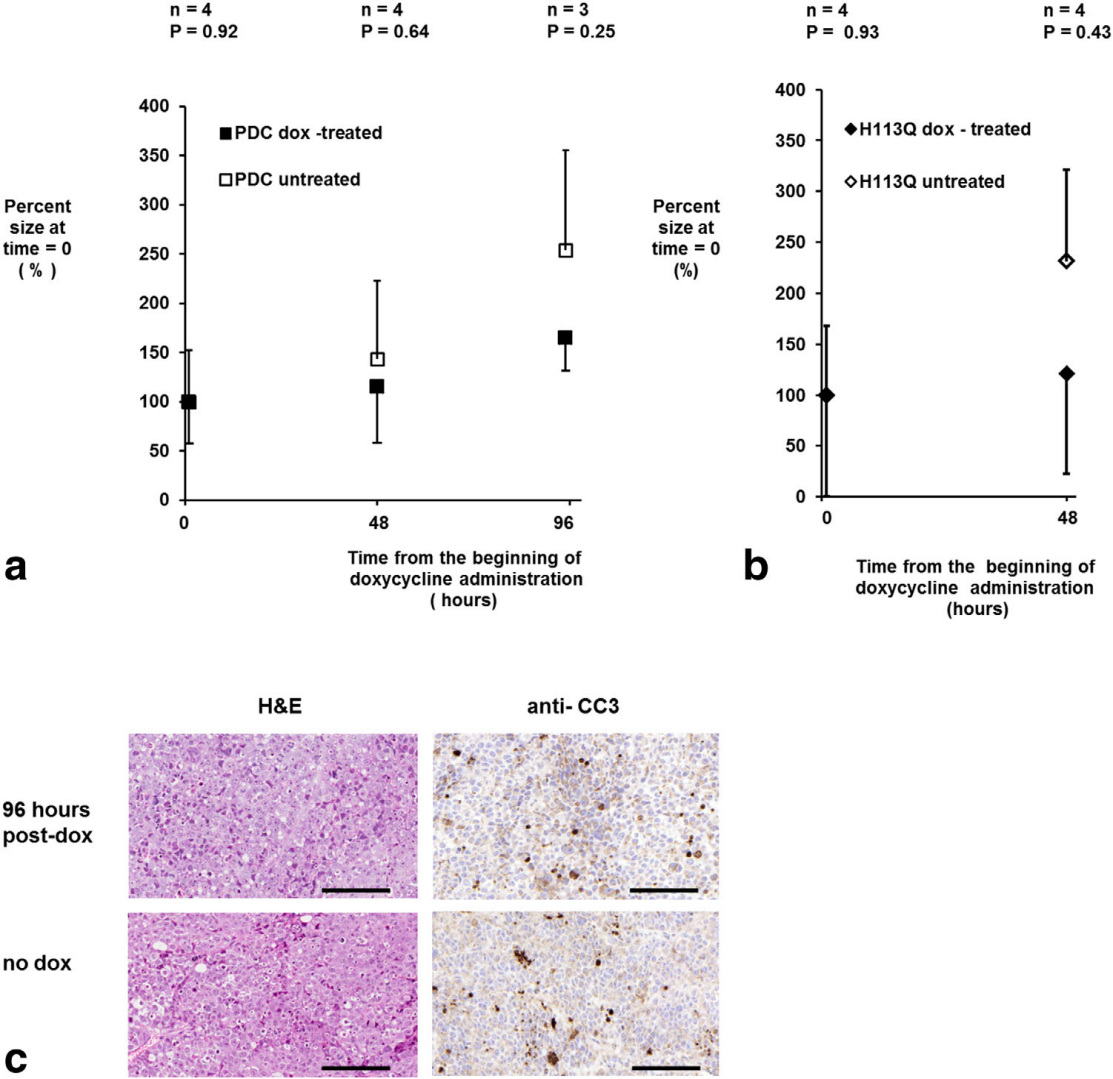
(a, b) Effect of induction of expression of wild‐type (a) and mutant PDC (b) on xenograft growth. The size of the xenografts derived from cells transfected with the tet‐on PDC or tet‐on H113Q constructs are expressed as the percentage of the volume measured at the beginning of doxycycline treatment (0 h). Caliper measurements of two xenograft diameters were made every 48 h from 21 d after implantation, when doxycycline was added to the drinking water as indicated. Euhus’ formula was used to calculate xenograft volume as described in the Methods section. Error bars represent one standard deviation of the mean. Only positive or negative error bars are shown for clarity. (**c**) Representative formalin‐fixed sections taken from xenografts grown for ∼21–24 d following subcutaneous implantation of cells transfected with the tet‐on PDC‐GFP‐V5/His construct. Where indicated, the mice were given 10 mg/mL doxycycline solution in their drinking water for 96 h prior to xenograft excision. The area of necrotic tissue was assessed via H&E staining, and apoptosis was investigated by probing with an anti‐CC3 antibody. Scale bars = 150 µm.

There was some evidence of a decrease in the growth rate of xenografts expressing the wild‐type (*zm*PDC‐GFP‐V5/His) and mutant enzymes (*zm*PDC/H113Q‐GFP‐V5/His) following doxycycline administration; however, this decrease was not statistically significant (Fig. 5a and 5b, respectively). The area of the tissue sections that comprised nonviable (necrotic and apoptotic) cells, as assessed by H&E staining, did not differ significantly between doxycycline‐treated and untreated xenografts (23.0 ± 6.6% and 21.5 ± 4.6%, respectively; SD, n = 4; *P* = 0.79), nor did the levels of apoptosis, as assessed by the levels of cleaved caspase 3 staining (14.3 ± 5.6% and 15.5 ± 8.4%, respectively; SD, n = 3; *P* = 0.85) (Fig. 4c).

**Figure 6:**
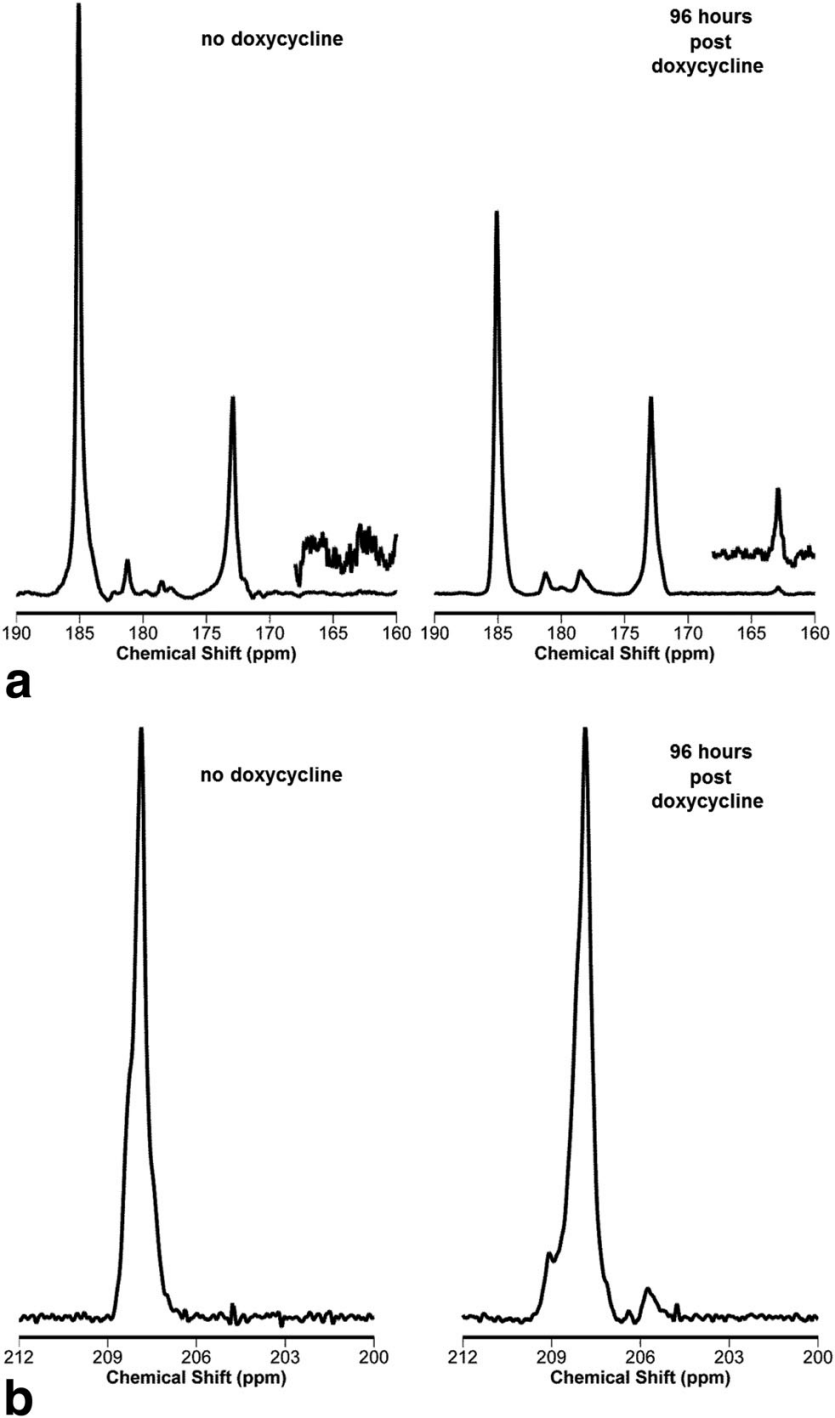
^13^C MRS detection of zmPDC activity in vivo following administration of hyperpolarized [1‐^13^C] pyruvate. (**a**) ^13^C spectra acquired from 8‐mm‐thick slices of xenografts ∼21 d after subcutaneous implantation HEK293T cells transfected with the PDC‐GFP‐V5/His construct. Experiments were performed either without doxycycline treatment (no doxycycline) or after 96 h of 10 mg/mL doxycycline solution administration (96 hours post doxycycline). A total of 10 μL/g body weight of a 75‐mM solution of hyperpolarized [1‐^13^C] pyruvate was injected over 2 s. A total of 128 single transient ^13^C spectra were acquired, starting 30 s from the injection (TR = 500 ms, nominal flip angle = 20°, SW = 6 kHz; delay from the middle of the excitation pulse to beginning of data acquisition was 900 µs). The insets show a 10× vertical scale expansion of the 162–168 ppm region of the resulting ^13^C spectra. Peak assignments: lactate (185 ppm), pyruvate hydrate (181 ppm), alanine (177 ppm), pyruvate (172.9 ppm), and bicarbonate (162.8 ppm). (**b**) Representative ^1^H‐decoupled spectra acquired from xenografts following injection of 10 μL/g body weight of 75 mM hyperpolarized [2‐^13^C] pyruvate. Spectra were acquired as described in panel a. Peak assignments: [2‐^13^C] pyruvate (207.4 ppm); the resonance at 205.6 ppm was tentatively assigned to [1‐^13^C] AcCoA.

Xenografts derived from cells transfected with *zm*PDC‐GFP‐V5/His, in which expression of the enzyme had been induced for 96 h by administration of doxycycline in the drinking water, showed approximately two‐fold higher bicarbonate/pyruvate signal ratios, calculated from the sum of the first 32 spectra acquired following intravenous injection of hyperpolarized [1‐^13^C] pyruvate, when compared with the uninduced controls (2.9 × 10^−2^ ± 1.1 × 10^−2^ [±SD], n = 7 and 1.5 × 10^−2^ ± 1.0 × 10^−2^ [±SD], n = 10, respectively; *P* = 0.016) (Fig. 6a). This increase was also observed when the bicarbonate signal was expressed relative to the lactate signal (1.3 × 10^−2^ ± 0.4 × 10^−2^ [±SD], n = 7 and 0.6 × 10^−2^ ± 0.5 × 10^−2^ [±SD], n = 10; *P* = 0.008) or the total observable ^13^C signal (7.0 × 10^−3^ ± 2.0 × 10^−3^ [±SD], n = 7 and 4.0 × 10^−3^ ± 2.0 × 10^−3^ [±SD], n = 10; *P* = 0.008). The total observable ^13^C signal included contributions from pyruvate, pyruvate hydrate, alanine, lactate, and bicarbonate. There was no measurable increase in the bicarbonate signal in xenografts expressing the inactive variant of *zm*PDC (tet‐on *zm*PDC/H113Q‐GFP‐V5/His) following doxycycline‐induction of enzyme expression. The bicarbonate/pyruvate signal ratio in untreated xenografts was 3.1 × 10^−2^ ± 3.1 × 10^−2^ (±SD, n = 3) and 2.3 × 10^−2^ (0.6 × 10^−2^ and 3.9 × 10^−2^, n = 2) after 96 h of doxycycline treatment. There was also no change when the bicarbonate signal was expressed relative to the total observable ^13^C signal, which was 6.0 × 10^−3^ ± 3.0 × 10^−3^ [±SD], n = 3) prior to treatment and 6.0 × 10^−3^ (2.0 × 10^−3^ and 10.0 × 10^−3^, n = 2) after 96 h of doxycycline treatment.

Because in many tissues there will be a background bicarbonate signal, we also investigated whether PDC activity could be assessed from the production of [1‐^13^C] acetaldehyde from [2‐^13^C] pyruvate. However, a [1‐^13^C] acetaldehyde resonance was not detectable in experiments using hyperpolarized [2‐^13^C] pyruvate in suspensions of PDC‐expressing cells (data not shown). In experiments on xenografts derived from these cells, a resonance at 209.4 ppm, corresponding to the resonance frequency of [1‐^13^C] acetaldehyde, was observed in some summed ^1^H ‐decoupled ^13^C spectra 96 h after doxycycline administration (five out of 10 experiments) (Fig. 6b). However, this resonance was also occasionally detected in uninduced tissue (two out of 10 experiments), indicating that the resonance was not specific to PDC activity. A resonance at 205.6 ppm, tentatively assigned to [1‐^13^C] acetyl‐coenzyme A (AcCoA) 23, was also detectable in some summed ^1^H ‐decoupled spectra acquired from xenografts, both before and after doxycycline administration (Fig. 6b). In those animals that showed this peak, its intensity, normalized to that of [2‐^13^C] pyruvate, increased approximately 1.9‐fold (1.9 ± 0.6; SD, *P* < 0.01) at 96 h after doxycycline administration.

## DISCUSSION

We have demonstrated that the pyruvate decarboxylase activity expressed by the *zm*PDC‐GFP‐V5/His fusion protein can be detected in vivo using hyperpolarized [1‐^13^C] pyruvate and ^13^C MRS and therefore that this system has the potential to be used as an MR gene reporter. The size of the *zm*PDC coding sequence (1.60 kbp), although considerably larger than that of GFP (0.73 kbp) and similar fluorescent reporter genes, is comparable to that of the widely used bioluminescence reporter, Firefly luciferase (1.65 kbp). A compact reporter size is an advantage when the payload constraints of delivery vectors are considered. Because it is a spectroscopic method in principle, it could be used with multiple gene reporters; for example, we have shown recently that the urea transporter can be used as a gene reporter in conjunction with hyperpolarized [^13^C]urea 24. The ^13^C resonances of urea, [1‐^13^C] pyruvate, and bicarbonate are resolved, therefore both reporters could be used in the same system. This is in general not possible with MRI methods that employ contrast agents modulating T_1_ or T_2_/T_2_* relaxation times, nor for positron emission tomography–based reporter genes 1, although there is this potential with chemical exchange saturation transfer (CEST)‐based MRI gene reporters 25.

Doxycycline‐induced expression of the *zm*PDC‐GFP‐V5/His fusion protein caused a decrease in cell growth rate in vitro (Fig. 2c). There was also some evidence for a decrease in growth rate in vivo of the xenografts derived from these cells following doxycycline administration in the drinking water (Fig. 5a, 5b). These effects on cell growth may be partially explained by the effects of doxycycline itself. Doxycycline, among other tetracyclines, is a potent inhibitor of mitochondrial translation, which inhibits formation of complexes I, III, IV, and V of the mitochondrial electron transfer chain 26, 27, 28, 29, leading to a decrease in citric acid cycle activity and, consequently, de novo pyrimidine synthesis 26. Consistent with this, we observed a decrease in the growth rate of untransfected wild‐type HEK293T cells in vitro after the addition of doxycycline to the growth medium (Fig. 2c). This growth inhibition was more pronounced, however, in cells expressing the *zm*PDC‐GFP‐V5/His transgene when compared with wild‐type cells (Fig. 2c), suggesting that growth could also be inhibited by expression of PDC activity. However, the observation that expression of the inactive (H113Q) mutant of the enzyme 17 also inhibited cell growth (Fig. 2c) suggests instead that this is a nonspecific effect of high‐level protein expression rather than expression of PDC activity per se. Moreover, this may be due to the GFP component of the construct since this has, in some contexts, been shown to have a nonspecific toxic effect on expressing cells 30, 31. The absence of a significant increase in apoptosis and necrosis in the cell line–derived xenografts in vivo, as assessed by anti‐CC3 and H&E staining, respectively (Fig. 6c), indicates that *zm*PDC‐GFP‐V5/His expression has a cytostatic rather than a cytotoxic effect. Therefore, the reporter might be more appropriate for use in tissues with low cell turnover.

Cell lysis increased the rate of [2,2,2‐^2^H_3_] acetaldehyde production from [U‐^2^H_3_] pyruvate approximately five‐fold in cells expressing *zm*PDC, implying that detection of PDC activity from measurements of labeled bicarbonate production in vivo is limited by the rate of pyruvate entry into the cell. This suggests that the reporter would work better in cells with higher rates of pyruvate transport and that there would be little to be gained from increasing the expression level or specific activity of *zm*PDC (eg, by removing the GFP tag), at least in HEK293T cells.

The background hyperpolarized ^13^C bicarbonate signal observed in the experiments described here can be explained by the activity of mitochondrial pyruvate dehydrogenase (PDH), which was shown previously in the rat heart to lead to the production of labeled bicarbonate from hyperpolarized [1‐^13^C] pyruvate 32. An alternative pathway involving pyruvate carboxylase has been reported in isolated perfused mouse liver 33; however, HEK293T cells do not express this enzyme activity 34. The background bicarbonate signal is expected to be much less in tumor cells; for example, EL4 lymphoma tumors show no detectable hyperpolarized bicarbonate signal following injection of [1‐^13^C] pyruvate 18.

The tet‐on doxycycline‐inducible gene expression system used here allowed rapid and reversible gene expression by oral administration of doxycycline 35, 36, allowing each xenograft to be used as its own control, which was important in demonstrating a PDC‐dependent increase in labeled bicarbonate production above the background level of labeled bicarbonate. In principle, the increased bicarbonate production in these experiments could also have been due to an effect of doxycycline on the production of CO_2_ by mitochondrial PDH activity. However, this is unlikely, because doxycycline had no effect on CO_2_ production in wild‐type cells, where the bicarbonate/pyruvate signal ratio was unaffected by doxycycline addition and was similar to the ratio observed in untreated cells transfected with the inducible wild‐type PDC vector (Fig. 3d). Moreover, doxycycline, by inhibiting the mitochondrial respiratory chain, would likely cause an increase in the mitochondrial NADH/NAD^+^ ratio by decreasing the rate of NADH oxidation 37. An increase in mitochondrial NADH/NAD^+^ ratio would be expected to inhibit PDH 38, 39, leading to a decrease in CO_2_ and bicarbonate production rather than an increase. In a limited number of experiments with xenografts expressing the mutant enzyme, we were unable to demonstrate an increase in bicarbonate production following administration of doxycycline in the drinking water.

An alternative approach to circumvent the problem of background bicarbonate signal would be to use hyperpolarized [2‐^13^C] pyruvate and detect the signal from [1‐^13^C] acetaldehyde. However, we were unable to reproducibly detect [1‐^13^C] acetaldehyde, which may be explained by the short T_1_ of [1‐^13^C] acetaldehyde (approximately 11.0 s compared with 40.7 s in bicarbonate) as well as its close proximity to the much larger pyruvate signal. In addition, rapid metabolic conversion of [1‐^13^C] acetaldehyde to [1‐^13^C] acetate, and further to [1‐^13^C] AcCoA and [1‐^13^C] acetyl‐carnitine, has been observed previously in experiments with hyperpolarized [1‐^13^C] acetate in vivo 23, 40, which will decrease the steady state pool size of labeled acetaldehyde. Measurements of ^13^C label flux between hyperpolarized [1‐^13^C, U‐^2^H_5_] ethanol and [1‐^13^C] acetate in mouse liver showed no evidence of the [1‐^13^C, U‐^2^H_4_] acetaldehyde intermediate, despite the much longer T_1_ of the deuterated acetaldehyde, which was approximately 19 s. Again this was attributed to a low steady‐state concentration of the labeled intermediate 22. Because [1‐^13^C] acetaldehyde exists in equilibrium with the hydrated form, with the hydrate ^13^C resonance at 91.4 ppm (data not shown), this will further decrease the sensitivity of its detection by ^13^C MRS 41. The resonance detected in xenografts at 205.6 ppm with hyperpolarized [2‐^13^C] pyruvate is likely to be from [1‐^13^C] AcCoA produced by PDH activity, as has been observed previously in heart muscle following administration of hyperpolarized [2‐^13^C] pyruvate 42. However, this would not explain the increase in this signal observed following doxycycline administration in the experiments described here. A possible explanation for this is that there is an increase in [1‐^13^C] acetate formation resulting from aldehyde dehydrogenase 2–catalyzed oxidation of the [1‐^13^C] acetaldehyde produced by PDC and that this is then rapidly converted to AcCoA by thiokinase. Build‐up of AcCoA has been observed in alcoholic fatty liver disease, where acetate produced by mitochondrial aldehyde dehydrogenase 2 from ethanol‐derived acetaldehyde is converted to AcCoA, but its further metabolism via the citric acid cycle is limited due to product inhibition of isocitrate dehydrogenase and α‐ketoglutarate dehydrogenase by NADH produced during acetaldehyde oxidation 43.

In conclusion, we have demonstrated the feasibility of using *zm*PDC as an MR gene reporter when used with hyperpolarized [1‐^13^C] pyruvate. However, the levels of bicarbonate produced above background were low, which would make it challenging to use in conjunction with an imaging experiment. Increasing *zm*PDC expression, at least in the cells used here, is unlikely to improve the sensitivity of the reporter, because PDC activity appeared to be limited by pyruvate transport into the cell. However, the potential for increased temporal resolution resulting from rapid signal acquisition coupled with subsequent rapid decay of the hyperpolarized signals and the capability of this reporter to be combined with other spectroscopy‐based gene reporters in the same experiment merits further development of this and other enzyme‐based gene reporters for use with hyperpolarized ^13^C MRS.

## Supporting information


**Supporting Figure S1**. ^1^H STEAM spectrum acquired from a 1 cm^3^ voxel within the xenograft after intraperitoneal administration of [U‐^2^H_3_] pyruvate. The animals were given 10 mg/mL doxycycline in their drinking water for 72 h prior to the experiments. The inset shows an expansion of the 10–8 ppm region of the spectrum with the vertical scale expanded by 10. The spectrum is representative of two independent experiments. The assignments were made by comparison with published spectra (Gillies RJ, Morse DL. In vivo magnetic resonance spectroscopy in cancer. Ann Rev Biomed Bioeng 2005;7:287–326).
