# Supplementary material for: 13C magnetic resonance spectroscopy measurements with hyperpolarized [1‐13C] pyruvate can be used to detect the expression of transgenic pyruvate decarboxylase activity in vivo
Source: Magn Reson Med. 2015 Sep 21;76(2):391–401. doi: 10.1002/mrm.25879 (PMC5025726; doi:10.1002/mrm.25879)
Supplement: Supplementary file 1 — Supporting Figure S1. 1H STEAM spectrum acquired from a 1 cm3 voxel within the xenograft after intraperitoneal administration of [U‐2H3] pyruvate. The animals were given 10 mg/mL doxycycline in their drinking water for 72 h prior to the experiments. The inset shows an expansion of the 10–8 ppm region of the spectrum with the vertical scale expanded by 10. The spectrum is representative of two independent experiments. The assignments were made by comparison with published spectra (Gillies RJ, Morse DL. In vivo magnetic resonance spectroscopy in cancer. Ann Rev Biomed Bioeng 2005;7:287–326). [file MRM-76-391-s001.docx]

**Supporting Materials**

**S1. Vector construction**

The *Zymomonas mobilis* PDC coding sequence (GenBank: M15393.2, GI: 21465420) in a pPLZM vector backbone was a gift from Professor Finian Leeper, Cambridge.

The *zm*PDC coding sequence was cloned into the pEF6-V5/His mammalian expression vector (Life Technologies Ltd, Paisley, UK) and the GFP coding sequence was inserted in frame with PDC and V5/His, to create a DNA sequence encoding an approximately 93.3 kDa protein, containing the *zm*PDC monomer fused at the N-terminus with GFP followed by the V5/His tag. This was then cloned by PCR as an *AgeI/FseI* fragment into a tet-inducible vector called pBS-TRE3G-YFP-ZnF-rtTA3pA (using components from pTRIPZ (Clontech Laboratories Inc., Mountain View, CA)). To make the mutant enzyme a 736 base pair *zm*PDC/T349G synthetic gene fragment (guanine substituted for thymine at position 349) and flanked by *BamHI* and *NcoI* sites, was ordered from Geneart (Geneart Life Technologies Ltd., Loughborough, UK). The fragment was then cloned into the unique *BamHI* and *NcoI* sites of the tet-on PDC-GFP-V5/His plasmid to generate the tet-on PDC/H113Q -GFP-V5/His plasmid.

**S2. HEK293T cells.**

The cells were from the European Collection of Cell Cultures (Teddington, UK) and were genotyped in-house with the AmpFISTR Kit (Applied Biosciences, Life Technologies, Carlsbad, CA). They matched the American Type Culture Collection consensus genetic profile in 88%.

**S3. Western Blotting**

Protein concentration in cell lysates was estimated using the Bradford protein assay (Bio-Rad Laboratories, CA, US). Twenty μg of protein, diluted in 4x LDS Sample Buffer (Nu PAGE, Life Technologies Ltd., Paisley, UK) and heat – denatured, were loaded per well. Protein was separated by SDS gel electrophoresis (4-12% Tris/Glycine Bio-Rad Mini-PROTEAN TGX Precast Gels, Bio-Rad Laboratories, CA, U.S.). PageRuler Prestained Protein Ladder (Fermentas/ Thermo Fisher Scientific, MA, U.S) was used to estimate the apparent molecular weights of the proteins. After semi-dry transfer the PVDF membranes (Immobilion-P, Millipore, pore size 0.45 μm) were incubated in a blocking buffer (5% w/v Marvel dried milk in PBS with 0.05% Tween-20 (PBS-T)). This was followed by incubation with a primary antibody, a wash in PBS-T, and incubation with an appropriate secondary antibody. Chemiluminescence was visualised using the ECL Plus Western Blotting Detection System (GE Healthcare Ltd, Amersham, UK) and x-ray film (Fuji Europe, Dusseldorf, Germany). The antibodies were diluted in blocking buffer to concentrations recommended by the manufacturers. Antibodies used: Goat anti-V5 (ab95038, Abcam, Cambridge, UK), horseradish peroxidase (HRP) - linked Rabbit anti - goat (P0449, DAKO, Glostrup, Denmark), Rabbit anti-Actin (A2066), goat anti-rabbit IgG-HRP (SC-2004, Santa Cruz Biotechnology, CA, U.S.).

**S4. Dynamic Nuclear Polarization**

A prototype 3.35 T DNP polariser (Oxford Instruments, Abingdon, UK) was used for all *in vitro* experiments, whereas a Hypersense instrument was used for experiments *in vivo*. A sample of [1-^13^C] or [2-^13^C] pyruvic acid, containing 15 mM of the trityl radical, OX063 (GE Healthcare, Little Chalfont, UK) and 1.5 mM gadoteric acid (DOTAREM; Guebert, Roissy, France) was placed inside the polarizer. After cooling the sample to approximately 1.4 K, irradiation at 93.972 GHz (or 93.972 in the case of the Hypersense polarizer) was applied using a 100 mW microwave source for an average of 45 minutes. Six mL of a dissolution buffer (40 mM HEPES; 94 mM NaOH, 30 mM NaCl, 100 mg/L EDTA) was heated to 180°C and pressurised to 10 bar before dissolving the frozen sample.

**S5. Immunohistochemistry**

Tissues were de-waxed and rehydrated prior to staining on a Leica BondMax Autostainer. Immunohistochemistry was performed using the Intense R kit with additional avidin/biotin blocking using the Vector SP-2001 avidin/biotin blocking kit (Vector Laboratories, Inc., Burlingame, CA, US). Staining was developed with DAB, using copper enhancement (DAB Enhancer, AR9432; Leica). H&E staining was performed as follows: sections were deparaffinised in xylene and rehydrated through graded alcohols before staining with Harris’ Haematoxylin, differentiation in 2% acid alcohol and bluing in running tap water and further staining with eosin. Slides were dehydrated, cleared and mounted in DPX mounting medium. Antibodies used: rabbit monoclonal anti-CC3 (CS9664; Cell Signaling Technology, Inc. Danvers, MA, US), anti-V5 goat polyclonal (ab95038; Abcam, Cambridge, UK), anti-GFP chicken polyclonal (ab13970, Abcam, Cambridge,UK), biotinylated donkey anti-rabbit and biotinylated donkey anti-goat (711-065-152 and 705-065-147, respectively; Jackson ImmunoResearch Laboratories, Baltimore, PA, US).

For fluorescence *in situ* hybridisation (FISH) using pan-centromeric chromosome paints, the slides were incubated in a pepsin solution for 10 minutes at 37 ^o^ C. Sections were then fixed in 4 % paraformaldehyde before application of chromosome paints. For each slide, 10 μl of biotin-labelled Human Chromosome Pan-Centromeric paint 1695-F-01 (Cambio,UK) and 10 μl of fluorescein isothiocyanate (FITC)-labelled Mouse Chromosome Pan-Centromeric Paint 1697-MB-01 (Cambio, UK) were applied. The sections were then covered with a glass cover slip, sealed with a rubber cement and then placed in a humid chamber to hybridize overnight. After removing the cement, the 3-Step Biotin Painting Kit labelled with Texas Red (Cambio, UK) was used according to the manufacturer’s protocols. Sections were mounted in ProLong Gold Antifade Reagent with added DAPI (Invitrogen, UK) and allowed to develop in a dark room for 24 hours.

**S6. GFP fluorescence imaging**

Animals were anesthetized and fur removed from the vicinity of the xenografts to improve imaging sensitivity. Fluorescence images were acquired using an IVIS 200 series camera (Perkin-Elmer, Waltham, MA) with an exposure of 1 second and f-stop 2, excitation wavelengths between 445 and 490 nm, emission wavelengths between 515 and 575 nm. Images were analysed with Living Image software (Perkin-Elmer).

**S7.** **^1^H-NMRS measurements following [U-^2^H_3_] pyruvate administration *in vivo*.**

Animals with xenografts derived from tet-on PDC-GFP transfected cells (n = 2) that had been given 10 mg/mL doxycycline in their drinking water for 72 hours prior to the experiments were anaesthetized and immobilized as for the ^13^C MRS measurements. An intraperitoneal injection line was placed in the animal’s abdomen, which allowed repeated injections without removing the cradle holding the immobilized animal from the magnet. ^1^H STEAM experiments were performed at 9.4 T using a volume coil/surface coil transmit/receive pair. An approximately 1 mL voxel was located inside the tumor. Excitation pulses (1 ms, Shinnar-LeRoux optimized 90 degree pulse) were centered either at the acetaldehyde resonance frequency (9.7 ppm) or the water resonance frequency (reference spectrum). VAPOR water suppression and outer-volume suppression were employed. Pulse sequence delays were TR 2.5 s, TM 20 ms and TE 10 ms with 128 averages. Spectra were acquired from a voxel within the xenograft using the STEAM sequence before the first intraperitoneal injection of 0.5 M sodium [U-^2^H_3_] pyruvate solution in water and during the 64 minutes after the first injection. A total of 4 injections were performed in each experiment, with volumes of 400µl (first) and 200 µl (subsequent 3), with 5 minute intervals between individual injections.


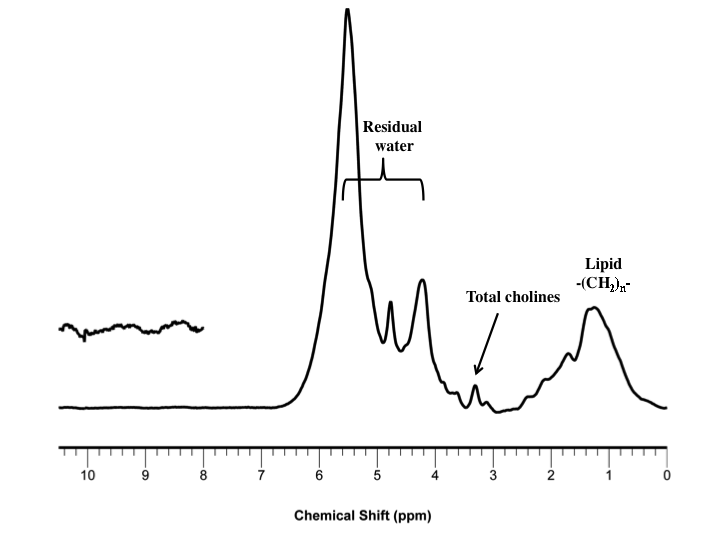


**Supporting figure S1**

A ^1^H STEAM spectrum acquired from a 1 cm^3^ voxel within the xenograft after intraperitoneal administration of [U-^2^H_3_] pyruvate. The animals were given 10 mg/ ml doxycycline in their drinking water for 72 hours prior to the experiments. The inset shows an expansion of the 10 – 8 ppm region of the spectrum with the vertical scale expanded by 10. The spectrum is representative of two independent experiments. The assignments were made by comparison with published spectra (1).

1. Gillies RJ, Morse DL. *In vivo* magnetic resonance spectroscopy in cancer. Ann Rev Biomed Bioeng 2005;7:287-326.
